# Prevalence and Determinants of Constipation in Community-Dwelling Adult Women in the Democratic Republic of Congo: A Cross-Sectional Study

**DOI:** 10.1007/s00192-025-06455-w

**Published:** 2025-12-02

**Authors:** Andy-Muller Luzolo Nzinga, Tara Reman, Denis Mukwege, Bernadette Mabenza Miangindula, Jeanne Bertuit, Veronique Feipel

**Affiliations:** 1 Laboratory of Functional Anatomy, Faculty of Human Motor Sciences, Université Libre de Bruxelles (ULB), Brussels, Belgium; 2 Pelvic Floor Rehabilitation Unit, University Clinics of Kinshasa (UNIKIN), Faculty of Medicine, University of Kinshasa, Kinshasa, Democratic Republic of Congo; 3 HESAV School of Health Sciences - Vaud, HES-SO University of Applied Sciences and Arts Western Switzerland, Av de Beaumont 21-1011, Lausanne, Switzerland; 4 Panzi General Referral Hospital, Evangelical University in Africa, Bukavu, Democratic Republic of Congo; 5 Department of Physical Medicine and Rehabilitation, Faculty of Medicine, University of Kinshasa, University Clinics of Kinshasa, Kinshasa, Democratic Republic of Congo; 6 Laboratory of Anatomy, Biomechanics and Organogenesis, Faculty of Medicine, Université Libre de Bruxelles, Brussels, Belgium

**Keywords:** Constipation, Women, Prevalence, Risk factors, Democratic Republic of Congo

## Abstract

**Introduction and hypothesis:**

Constipation is one of the most frequent functional gastrointestinal dysfunctions, with a negative impact on women’s quality of life. There are currently no epidemiological data on the magnitude of constipation among adult women in the Democratic Republic of Congo (DRC).

To determine the prevalence of constipation among adult women in the DRC and to identify the risk factors associated with it.

**Methods:**

This was a community-based cross-sectional study conducted from 2021–2023 among 516 adult women (≥18 years) in six provinces of the DRC. A multistage, geographically and ethnolinguistically stratified sampling approach was used. Pregnant or postpartum women ≤ 6 months, survivors of sexual violence and those with anorectal and rectovaginal fistulas were excluded. The diagnosis of constipation was made for a score of ≥ 11 on the KESS constipation questionnaire. Pelvic floor muscles were assessed according to the PERFECT scheme. Binary logistic regression was performed to identify risk factors.

**Results:**

The prevalence of constipation was 16.1% (95% CI 13.1–19.6%), with a higher prevalence observed among women over the age of 50. Hard stool consistency and difficult, painful evacuating were the most common suggestive symptoms encountered in constipated women (*p* < 0.001). Constipation negatively affected sexual quality of life (*p* < 0.001). In the multivariable analysis, lower mental health scores of life quality (adjusted odds ratio [aOR] 0.98; 95% CI 0.97–0.99) and belonging to the Baluba ethnolinguistic group (aOR 0.31; 95% CI 0.13–0.68) were protective factors against.

**Conclusion:**

Constipation is a non-negligible health concern among adult women in the DRC.

## Introduction

Constipation is one of the most common functional gastrointestinal dysfunctions, with a negative impact on women’s quality of life. When it becomes chronic, it constitutes a considerable economic and social burden and is therefore considered a common health problem worldwide [1–3]. It is generally manifested by persistent difficulty in evacuating stools, a feeling of incomplete bowel evacuation and/or infrequent bowel movements (less than three times a week) in the absence of alarm symptoms or secondary causes [4, 5]. In practice, its definition may vary according to several factors, including the underlying mechanism, the patient’s perception, its impact on quality of life, and the criteria used to make the diagnosis (self-report, clinical criteria, validated scales) [6].

From a pathophysiological standpoint, the American Gastroenterological Association (AGA) distinguishes three subtypes of constipation, which are often interrelated. (a) Transit constipation (or slow transit constipation) associated with slow colonic transit due to colonic neuromuscular dysfunction. (b) Obstructed defecation (or dyschezia) marked by straining to defecate and a sensation of blockage during evacuating, generally secondary to abnormalities of the pelvic floor or rectum. (c) Normal transit constipation (or functional constipation), the most common, characterized by normal transit and a pelvic floor with functional integrity [6, 7]. This complexity of definitions and symptoms impacts on efforts to standardize epidemiological criteria, leading to wide variations in prevalence estimates worldwide [4, 5]. Its prevalence in women varies widely, from 10.2% to 63%, depending on the populations studied and the diagnostic tools employed [8–11].

Constipation is a multifactorial condition resulting from a complex of interconnected various factors, including physiological (age, gender, intestinal motility disorders, hormonal imbalances), psychological, environmental, dietary (low-fiber diet, dehydration), behavioral (sedentary lifestyle, low physical activity), pharmacological, as well as social and neurological factors [1, 12, 13].

Despite recognition of its existence and high prevalence in the literature, there are currently no epidemiological data on the magnitude of constipation among adult women in the Democratic Republic of Congo (DRC).

Moreover, in our clinical practice, few women consult a specialist for constipation as a first-line treatment, as some women consider it to be trivial, while others adopt personal strategies to manage the condition. These include self-medication, the use of local herbal remedies whose effects on the body are not well known, frequent enemas with water and other products such as ginger and chilli, which may further affect the rectum, the adoption of a diet or even the use of digital manoeuvres, sometimes to no avail. In this way, constipation quietly settles into their daily lives, silently affecting them to the point of impairing their quality of life.

Moreover, in clinical practice within our settings, few women seek medical care primarily for constipation. Some women consider it to be trivial, while others adopt personal strategies to manage the condition. These may include self-medication, the use of traditional herbal remedies with unknown effects on the body, frequent water enemas or with some substances such as ginger or chili, which may further damage the rectum. Others modify their diet or resort to rectal or vaginal digitation, which are sometimes ineffective. Thus, constipation gradually becomes a silent part of their daily lives, affecting them quietly until it eventually compromises their quality of life.

This study was conducted within this perspective, with the objectives of determining the prevalence of constipation among adult women in the Democratic Republic of Congo (DRC) and identifying its associated risk factors. The goal is to provide robust epidemiological and clinical data to guide healthcare professionals and policymakers in developing appropriate management strategies for this often-neglected problem.

## Materials and Methods

### Ethical Considerations

Ethical approval was obtained under number 423/CNES/BN/PMMF/2021. All participants provided written informed consent prior to data collection.

### Study Design, Setting and Participants

This community-based cross-sectional study was conducted from 2021 to 2023 among adult women (≥18 years) residing in six provinces of the Democratic Republic of Congo (DRC), selected to represent the ethnolinguistic diversity of the country [14]. A multistage, geographically and ethnolinguistically stratified sampling approach was used to recruit women from the community. The first stage of the selection process involved grouping the regions of the DRC according to their ethnolinguistic characteristics to select one province per region (the Baluba, Bakongo, Bangala, Swahili and Cosmopolitan “Kinshasa” zones). The second stage involved selecting a city in each province, while the third stage involved selecting a health zone in each city. Recruitment was carried out in Kasaï Orientale province, where Tshiluba is spoken by the Baluba community; Equateur, where Lingala is spoken by the Bangala; Nord-Kivu and Sud-Kivu, where Swahili predominates, and Kongo-Central, where Kikongo is spoken by the Bakongo. The City-Province of Kinshasa, a cosmopolitan area where Lingala is widely spoken by the Kinois population, was also included. Women from the North and South Kivu provinces were grouped as the Swahili group. All selected health zones were urban, except for that of Kongo-Central, which features both urban and rural characteristics. Owing to time constraints, limited financial resources and accessibility challenges, this study did not include women from rural areas. Community health workers helped raise awareness about recruitment within the community. In each selected health zone, they used a door-to-door approach for recruitment. After a thorough explanation of the nature of the study, women who agreed to participate then attended the selected health centre for the interview and examination.

On the basis of a reported constipation prevalence of 28.5% among African women in the study of Séhonou et al. [15], the required sample size was calculated at 313 using the formula: *n* = z^2^ × p(1 − p)/i^2^, where *n* is the required sample size, *z* = 1.96 corresponds to the 95% confidence level, *p* is the expected prevalence based on previous literature, and *i* = 0.05 represents the desired precision (or margin of error) [16].

A total of 516 apparently healthy adult women out of 519 recruited were included in the study. Participants were required to speak French, or one of the national languages, including Lingala, Tshiluba, Kikongo or Swahili. If needed, translation was provided by a trained healthcare professional fluent in one of these languages. Pregnant women, women within 6 months of delivery, survivors of sexual violence and women with anorectal fistulas were excluded from the study. The exclusion of women with these characteristics was justified, as they represent specific populations in which the prevalence of constipation tends to be higher.

### Outcomes

The questionnaire was carefully completed by the examiner. Sociodemographic and clinical data were collected, including age, residential setting, ethnic group, education level, marital status, occupation, body mass index (BMI), menopausal status, delivery, parity, gravidity, episiotomy and perineal tears. A vulvar examination and assessment of the pelvic floor muscles by vaginal examination were used to collect variables such as vaginal elasticity, pelvic floor muscle (PFM) relaxation and PFM tone [17]. These examinations were performed by two healthcare professionals specialized in perineal rehabilitation. No anorectal examination was performed.

Constipation was assessed using the Knowles Eccersley Scott Symptom (KESS) constipation scale, a validated tool developed to facilitate the diagnosis of constipation. Its content is based on the Rome II diagnostic criteria. The tool consists of 11 items covering duration of constipation, use of laxatives (with or without suppositories or enemas), performance of digitation manoeuvres, frequency of bowel movements and dyschezia. Each item offers four to five response options on the Likert scale, with scores ranging from 0 to 3 or 4 points. The total KESS score is the sum of the scores for each item, with a maximum possible score of 39. A score of 0 indicates the absence of symptoms, while a higher score indicates greater symptom severity. The diagnosis of constipation is retained for a threshold ≥ 11. Any symptom of constipation occurring at least occasionally, in 25% or more of bowel movements, was considered pathological [18].

The Medical Outcomes Study 36-item Short-Form Health Survey (SF-36) is a generic scale most used to measure quality of life in the general population. It assesses physical and mental health, with scores ranging from 0 (worst) to 100 (best) [19].

### Statistical Analysis

Data were collected and recorded using REDCap (Research Electronic Data Capture), a secure web application designed for research database management. Outliers and data entry errors were cleaned using Microsoft Excel version 2024. Statistical analyses were performed using R Studio version 4.4.1 for Windows. Missing data representing less than 5% were treated by simple imputation (by mean or median, as appropriate). For modelling purposes, observations with more than 10% missing data were excluded, and a sensitivity analysis was performed to estimate the potential biases associated with this exclusion.

Descriptive statistics were calculated according to the nature and normal distribution of the variables. The normality of continuous variables was assessed using the Shapiro–Wilk test. Normally distributed continuous quantitative variables were summarized as mean ± standard deviation (SD), while non-normally distributed or discrete quantitative variables were presented as median with interquartile range (IQR). Categorical variables were expressed as frequencies and percentages (%). 95% confidence intervals (CIs) were calculated for prevalences to take account of sampling fluctuation. The threshold for statistical significance in inferential analyses was set at 5%. Characteristics of the group of women with constipation were compared with those without constipation. Normally distributed quantitative variables were compared using the student’s *t*-test, while those that did not follow a normal distribution were analysed using the Wilcoxon test. Categorical variables were compared using Pearson’s chi-square test (or Fisher’s exact test, if necessary). In cases of a significant overall test, post-hoc comparisons between groups were performed, and *p* values were adjusted using the Holm method to control the risk of type I error associated with multiple comparisons.

A univariate binary logistic regression was first performed to explore the association between each independent variable and the dependent variable (constipation). These associations were represented as crude odds ration (COR) with 95% confidence interval (95% CI). Variables with a *p* value ≤ 0.25 in the univariable analysis, and those not significant but deemed relevant according to the literature, were included in the multivariable logistic regression model to ensure that no important predictors were omitted. The enter method was applied, and multiple models were generated. The final model was selected on the basis of the lowest Akaike information criterion (AIC). Multicollinearity was assessed using the variance inflation factor (VIF). Variables with a VIF greater than 2.5 were considered to have suspect collinearity.

In these cases, one of the correlated variables was excluded from the model. The goodness-of-fit of the model was assessed using the Hosmer–Lemeshow test. Associations from multivariable analysis were reported using adjusted odds ratios (aOR) with 95% CI.

## Results

Recruitment in six provinces of the DRC resulted in the inclusion of 519 women. Three women were excluded due to the presence of a rectovaginal fistula, bringing the final number of participants to 516 (99.4%). Of these 516 women, 110 (21.3%) did not accept a digital vaginal examination. These women presented significantly different characteristics (younger age, fewer married women, a higher proportion of nulliparous and primiparous women, and lower levels of education) than those who accepted the examination (*p* < 0.005). The main reasons for not undergoing the examination included: personal concerns, being older than the examiner, the examiner being male, or cultural beliefs, currently menstruating and virginity. Small proportions of missing values were identified and imputed by the mean or median, depending on the distribution characteristics of the variables concerned. These included age (*n* = 4; 0.8%) and body mass index (*n* = 2; 0.4%).

### Study Population and Baseline Characteristics

Table 1 shows that the mean age and BMI of the women were 33.4 ± 14.5 years and 22.7 ± 4.3 kg/m^2^, respectively. Age ranged from 18 to 85 years. The median KESS score for constipation, physical health score, and mental health score were 1.5, 87.5, and 80.7, respectively. The sample was predominantly composed of women with an education level below secondary (60.7%), living mainly in urban areas (78.7%), and 23.1% were married or cohabiting. Additionally, 51.2% were unemployed, 77.9% had given birth, with 35.5% grand multiparous (≥ 5) and 18% were postmenopausal.

**Table 1:** Comparison of sociodemographic and clinical characteristics between groups of women with and without constipation

Variables	Total		No constipation		Constipation		*p* value
	*N*	Values	*n*	Values	*n*	Values	
Age (years), mean ± SD	516	33.4 ± 14.5	433	32.7 ± 14.1	83	37.1 ± 16.2	***0.012***
BMI (kg/m^2^), mean ± SD	516	22.7 ± 4.3	433	22.8 ± 4.3	83	22.8 ± 4.4	0.98
Gravidity, median (IQR)	516	3 (1–6)	433	3 (1–6)	83	3 (1–6.5)	0.59
Parity, median (IQR)	516	3 (1–6)	433	3 (1–6)	83	2 (1–6)	0.87
Episiotomy, médiane (IQR)	516	0 (0–1)	433	0 (0–1)	83	0 (0–1)	0.81
Perineal tears, median (IQR)	516	0 (0–0)	433	0 (0–0)	83	0 (0–0)	0.35
KESS score, median (IQR)	516	1.5 (0–8)	433	0 (0–4)	83	14 (11–17)	**<*0.001***
Physical health score (SF-36), median (IQR)	516	87.5 (70–86)	433	88.3 (72.7–95.8)	83	80 (41–93.8)	**<*0.001***
Mental health score (SF-36), median (IQR)	516	80.7 (65–92)	433	83 (66.8–92.8)	83	70.1 (50.4–88)	**<*0.001***
Residential setting							
Urban		406 (78.7)		342 (79)		64 (77.1)	0.70
Urbano-rural		110 (21.3)		91 (21)		19 (22.9)	
Menopausal status							
No		419 (81.2)		356 (82.2)		63 (75.9)	0.17
Yes		97 (18.8)		77 (17.8)		20 (24.1)	
Education level							
≥Secondary		203 (39.3)		172 (39.7)		31 (37.3)	0.68
<secondary		313 (60.7)		261 (60.3)		52 (62.7)	
Marital status							
Others		242 (46.9)		198 (45.7)		44 (53)	0.22
Married/cohabiting		274 (53.1)		235 (39)		39 (47)	
Occupation status							
Unemployed		252 (48.8)		214 (49.4)		38 (45.8)	0.54
Employed		264 (51.2)		219 (50.6)		45 (54.2)	
Delivery							
No		114 (22.1)		95 (21.9)		19 (22.9)	0.84
Yes		402 (77.9)		338 (78.1)		64 (77.1)	
Parity group							
Nulliparous (zero)		114 (22.1)		95 (21.9)		19 (22.9)	0.63
Primiparous (one)		84 (16.3)		69 (15.9)		15 (18.1)	
Multiparous (2 to 4)		135 (26.2)		118 (27.3)		17 (20.5)	
Grand multiparous (≥ 5)		183 (35.5)		151 (34.9)		32 (38.6)	
Episiotomy		150 (29.1)		125 (28.9)		25 (30.1)	0.81
Perineal tears		84 (16.3)		73 (16.9)		11 (13.3)	0.41
PFM relaxation		250 (61.6)		207 (61.8)		43 (60.6)	0.54
PFM tone at 3 o’clock							
Normal		191 (74)		157 (46.9)		34 (47.9)	0.56
Decreased tone		110 (27.1)		94 (28.1)		16 (22.5)	
Increased tone		105 (25.9)		84 (25.1)		21 (29.6)	
PFM tone at 9 o’clock							
Normal		192 (47.3)		160 (47.8)		32 (45.1)	0.43
Decreased tone		112 (27.6)		95 (28.4)		17 (23.9)	
Increased tone		102 (25.1)		80 (23.9)		22 (31)	
Vaginal elasticity							
No		107 (26.4)		86 (25.7)		21 (29.6)	0.49
Yes		299 (73.6)		249 (74.3)		50 (70.4)	

*BMI* body mass index, *IQR* interquartile range, *SD* standard deviation, *PFM* pelvic floor musle

Comparing women with and without constipation, Table 1 shows that those with constipation were significantly older (37.1 ± 16.2 years vs. 32.7 ± 14.1 years; *p* = 0.012), and had significantly higher KESS scores, indicating more severe constipation (14 vs. 0; *p* < 0.001) than those without constipation. They also exhibited a lower quality of life, both in terms of physical health (80.0 vs. 88.3; *p* < 0.001) and mental health (70.1 vs. 83.0; *p* < 0.001), compared to women without constipation. PFM characteristics, gynecological and obstetric histories, as well as other sociodemographic features, were similar between the two groups (*p* > 0.05) (Table 1).

### Prevalence of Constipation and its Symptoms

Of the 516 women included in the study, 83 (16.1%; 95% CI 13.1–19.6%) experienced constipation according to the KESS scale (cutoff ≥ 11/39) (Table 2). This condition was significantly more frequent in women aged over 50 (27.3%; 95% CI 17.3–37.2%; *p* = 0.017) and less frequent in the Baluba group (7.3%; 95% CI 2.4–12.1%; *p* < 0.011), compared with other ethnolinguistic groups (Table 3).

**Table 2:** Prevalence of constipation and its symptoms according to the KESS scale

Variables	*n* (Total)	Prevalence	95% CI
Constipation (KESS scale)	83 (516)	16.1	13.1–19.6
Symptoms of constipation			
Duration of constipation > 18 months	62 (516)	12	9.4–15.2
Frequency of bowel movements ≤ 2 times a week	53 (516)	10.3	7.8–13.3
Straining to defecate (≥25% defecation time)	183 (516)	35.5	31.3–39.9
Feeling of incomplete bowel evacuation (≥25% defecation time)	127 (516)	24.6	21–28.6
Abdominal pain (≥25% defecation time)	92 (516)	17.8	14.6–21.5
Enemas or digitation (≥25% defecation time)	51 (516)	9.9	7.5–12.9
Time in the toilet > 5 min	112 (516)	21.7	18.2–25.6
Pain during straining/defecation (≥25% defecation time)	161 (516)	31.2	27.2–35.4
Hard stools (≥25% defecation time)	226 (516)	43.8	39.4–48.2
False urge to defecate	115 (516)	22.3	18.8–26.2

**Table 3:** Prevalence of constipation according to women’s characteristics

Variables, *n *(%)	*n* (Total)	Prevalence	95% CI
Age group (years)			
18–24	25 (193)	12.9^a^	8.2–17.6
25–49	37 (246)	15^a^	10.5–19.5
≥50	21 (77)	27.3	17.3–37.2
*p* value	***0.017***		
*P* value^a^	0.62		
Residential setting			
Urban	64 (406)	15.8	12.2–19.3
Urbano-rural	19 (110)	17.3	10.2–24.3
*p* value	0.70		
Ethnic group			
Kinois	16 (56)	28.6*	16.8–40.3
Swahili	19 (120)	15.8	9.2–22.4
Bakongo	19 (110)	17.3	10.2–24.3
Bangala	21 (120)	17.5	10.6–24.3
Baluba	8 (110)	7.3*	2.4–12.1
Kinois vs Baluba			
*p* value (Ethnic group)	***0.011***		
*p* value* (Kinois vs Baluba)	***0.005***		
Menopausal status			
No	63 (419)	15	11.6–18.5
Yes	20 (97)	20.6	12.5–28.7
*p* value	0.17		
Parity group			
Nulliparous (zero)	19 (114)	16.7	9.8–23.5
Primiparous (one)	15 (84)	17.9	9.6–26.0
Multiparous (2 to 4)	17 (135)	12.6	7.0–18.2
Grand multiparous (≥5)	32 (183)	17.5	11.9–22.9
*p* value	0.63		

Among the suggestive symptoms of constipation, the most frequently reported by women was the presence of hard stools, with a prevalence of 43.8% (95% CI 39.4–48.2%), followed by straining during defecation (35.5%; 95% CI 31.3–39.9%), and experiencing pain during straining/defecation (31.2%; 95% CI 27.2–35.4%). A frequency of bowel movements less than or equal to twice a week was reported by 10.3% of women (95% CI 7.8–13.3%) (Table 2).

### Comparison of Constipation Symptoms Between Women Who Experienced and Did Not Experience Constipation

Table 4 shows a comparison of digestive symptoms suggestive of constipation between women with and without constipation. It shows that women who experienced constipation were significantly more exposed to several digestive symptoms including hard stool consistency, pain during straining/defecation, feeling of incomplete bowel evacuation, as well as straining during defecation, compared to those who did not experienced constipation. They also had a higher proportion of infrequent bowel movements (≤2 times per week). Furthermore, the use of enemas or digitation to facilitate stool evacuation was more frequent in this group. Among sexually active women, those who experienced constipation reported a greater impact on their sexual quality of life (Table 4).

**Table 4:** Comparison of constipation symptoms between groups of women with and without constipation

Variables, *n *(%)	No constipation *N* = 433	Constipation *N* = 83	*p* value
Duration of constipation			
≤18 months	413 (95.4)	41 (49.4)	<0.001
>18 months	20 (4.6)	42 (50.6)	
Frequency of bowel movements			
>2 times a week	416 (96.1)	47 (56.6)	<0.001
≤2 times a week	17 (3.9)	36 (43.4)	
Straining to defecate^a^			
No	329 (76)	4 (4.8)	<0.001
Yes	104 (24)	79 (95.2)	
Feeling of incomplete bowel evacuation^a^			
No	377 (87.1)	8 (9.6)	<0.001
Yes	56 (12.9)	75 (90.4)	
Abdominal pain^a^			
No	389 (89.8)	35 (42.2)	<0.001
Yes	44 (10.2)	48 (57.8)	
Enemas or digitation^a^			
No	410 (94.7)	55 (66.3)	<0.001
Yes	23 (5.3)	28 (33.7)	
Time in the toilet > 5 min			
≤5 min	381 (88)	23 (27.7)	<0.001
>5 min	52 (12)	60 (72.3)	
Pain during straining/defecation^a^			
No	351 (81.1)	4 (4.8)	<0.001
Yes	82 (18.9)	79 (95.2)	
Stools consistency^a^			
Normal	289 (66.7)	1 (1.2)	<0.001
Hard	144 (33.3)	82 (98.8)	
False urge to defecate			
No	375 (86.6)	26 (31.3)	<0.001
Yes	58 (13.4)	57 (68.7)	
Impact on sexuality among sexually active women			
No	230 (95)	37 (78.7)	<0.001
Yes	12 (5)	10 (21.3)	

^a^(≥25% defecation time)

### Factors Associated with Constipation

Univariate binary logistic regression analysis (Table 5) revealed that age (COR 1.02; IC95% 1.01–1.03), physical health score (COR 0.98; IC95% 0.97–0.99), mental health score (COR 0. 98; IC95% 0.97–0.99) and membership of the Baluba ethnolinguistic group (COR 0.34; IC95% 0.14–0.70) were factors associated with the occurrence of constipation.

**Table 5:** Factors associated with constipation: univariable and multivariable analyses

Factors	COR (95% CI)	*p* value	aOR (95% CI)	*p* value
Age (years)	1.02 (1.01–1.03)	***0.013***	1.01 (0.98–1.03)	0.28
BMI (kg/m^2^)	1.00 (0.94–1.05)	0.98	0.95 (0.90–1.01)	0.16
Physical health score (SF-36)	0.98 (0.97–0.99)	**<*0.001***	**–**	**–**
Mental health score (SF-36)	0.98 (0.97–0.99)	**<*0.001***	0.98 (0.97–0.99)	**<*0.001***
Ethnic group				
Others	*1*		*1*	
Baluba	0.34 (0.14–0.70)	***0.006***	0.31 (0.13–0.68)	***0.005***
Menopausal status				
No	*1*			
Yes	1.46 (0.82–2.53)	0.17	–	–
Statut marital				
Others	*1*			
Married/cohabiting	0.74 (0.46–1.19)	0.22	–	–
Education level				
≥Secondary	*1*		*1*	
<Secondary	1.10 (0.68–1.80)	0.68	1.25 (0.71–2.25)	0.43
Occupation status				
Unemployed	*1*		1	
Employed	1.15 (0.72–1.86)	0.54	1.01 (0.60–1.68)	0.95
Residential setting				
Urban	*1*			
Urbano-rural	1.11 (0.62–1.92)	0.70	–	–
Parity	1.01 (0.95–1.09)	0.58	0.96 (0.86–1.07)	0.52
Episiotomy				
No	*1*		1	
Yes	1.06 (0.62–1.75)	0.81	1.05 (0.60–1.80)	0.85
Perineal tears				
No	*1*			
Yes	0.75 (0.36–1.43)	0.41	–	–

Hosmer–Lemeshow test (*p* = 0.20)

*COR* crude odds ratio, *BMI* body mass index, *aOR* adjusted odds ratio

However, other variables, such as BMI, menopause status, marital status, education level, occupation, residential setting, parity, episiotomy and perineal tears, were not found to be significantly associated with constipation in this univariable analysis.

In the multivariable analysis (Table 5), after adjusting for other potential confounding factors, mental health score (aOR 0.98; 95% CI 0.97–0.99) and belonging to the Baluba ethnolinguistic group (aOR 0.31; 95% CI 0.13–0.68) remained significantly associated with constipation. These findings suggest that women with better mental health scores (quality of life) and those from the Baluba ethnolinguistic group were less likely to experience constipation.

## Discussion

The prevalence of constipation among adult women in the DRC, estimated at 16.1% (95% CI 13.1–19.6%), is in the low range of rates reported worldwide. These rates vary considerably, from 10.2% to 63%, depending on the diagnostic criteria used and the populations studied [9–12].

Barberio et al. [20] observed that the pooled prevalence of functional constipation tends to decrease with successive updates of the Rome diagnostic criteria, being 26.1% (95% CI 6.5–52.9%) for Rome I, 18.1% (95% CI 11.1–26.4%) for Rome II and 17.3% (95% CI 9.0–27.6%) for Rome III. In studies using the Rome II criteria, as in the present study, the prevalence of constipation ranged from 18.1% to 33.2% [20–22]. The rate observed among adult women in the DRC also falls within a range comparable to that reported in these studies. It also corroborates the pooled prevalence rate of 17.4% (95% CI 13.4–21.8%) reported by Suares et al. [11] in their systematic review of general population studies. Data on the prevalence of constipation in women in the African region are scarce [20]. The study by Séhonou et al. [15], carried out in Benin, reports a very high prevalence of 28.5%, diagnosed according to the Rome VI criteria, compared with the present study. Collete et al. [8] note that in Brazil, the prevalence of constipation appears to be high in individuals with black skin and in low-income groups.

The prevalence of constipation was particularly high among women over 50, reaching 27.3% (95% CI 17.3–37.2%), with a statistically significant association between advanced age and the occurrence of constipation. These findings are consistent with the literature [6, 11, 23, 24].

In the present study, the most frequently reported gastrointestinal symptoms, with greater risk of developing constipation, point more towards the subtypes of functional constipation and obstructed defecation (dyschezia). Symptoms such as hard stools, straining during defecation, pain during straining/defecation and a feeling of incomplete evacuation have been identified as major clinical manifestations of constipation, as also demonstrated by the studies of Séhonou et al. [25] and Podzemny et al. [26]. This trend is confirmed by the literature, which indicates that the most common subtype of constipation in women is functional constipation [6]. It should be noted that there is significant overlap between the different subtypes of constipation, and isolated symptoms often fail to distinguish them clearly [6, 27, 28].

Several factors were individually associated with constipation. However, after adjusting for certain confounding factors and accounting for multicollinearity in the multivariable analysis, two factors remained significantly associated with a reduced risk of constipation: a better mental health score (aOR 0.98; 95% CI 0.97–0.99) and belonging to the Baluba ethnolinguistic group (aOR 0.31; 95% CI 0.13–0.68).

Overall, these results suggest that socio-cultural factors and those related to general well-being play a key role in the occurrence of constipation, in particular, physical health, lifestyles and dietary habits specific to certain ethnolinguistic groups. It could be that women from the Baluba group may have a high-fiber diet, better hydration, or more adapted lifestyles and daily physical activity, as well as a health status favouring good intestinal and pelvic floor muscle function, making them less constipated. Future studies that take these factors into account may help to verify this hypothesis.

This study also highlights that poor mental health is a predictive factor for constipation. The deterioration in mental health, as assessed by the general quality of life scale, may reflect a psychologically vulnerable state influenced by various health and socio-economic factors to which these women are exposed.

However, due to the cross-sectional design of this study, it is not possible to determine the direction of this relationship: it remains unclear whether constipation contributes to poor mental health, or conversely, whether a compromised psychological state promotes the onset of constipation in these women.

The literature emphasizes that constipated patients often suffer from psychological disorders associated with stressful life events such as anxiety, depression and physical or sexual abuse. According to other authors, chronic constipation, particularly when accompanied by dyssynergic defecation, is frequently linked to major psychological disorders [12, 13, 26].

In addition to psycho-emotional factors, certain contributors not explored in this study, such as pelvic organ prolapse, anorectal prolapse, or functional causes like anismus or defecatory dyssynergia [26, 29], could also contribute to obstructed defecation (dyschezia) in the surveyed women.

It is important to emphasize that constipation is a multifactorial condition. According to current recommendations (grade B), multiple risk factors should be systematically assessed, including age, multiple medications, certain diets, psychiatric disorders, and a history of physical or sexual abuse [7, 30]. However, not all of these dimensions were investigated in the present study, although participants who reported a history of sexual violence were excluded from the analysis. It remains difficult to determine the exact influence of some factors on constipation. Furthermore, the literature reveals a lack of consensus regarding certain key contributors to this condition [12, 13].

Targeted and appropriate management of constipation is essential to prevent its complications and its impact on women’s quality of life. In this study, many women frequently resorted to enemas or vaginal/rectal digitation to evacuate their stools, yet these methods often failed to provide lasting relief. Moreover, among those who were sexually active, constipation had a negative impact on their sexual life. According to Vitton et al. [6], this impact, particularly in women with obstructed defecation, should not be overlooked, as it may lead to issues such as dyspareunia.

Given the cross-sectional design of this study, the observed relationship between these potential predictors and constipation does not imply causality. Future research should expand on these findings, focusing on more specific analytical and clinical approaches to the diagnosis of constipation, and highlight existing causes among women affected by constipation in this socio-cultural context of the DRC. This study should be complemented by studies using the Rome IV criteria, to better exclude the diagnosis of irritable bowel syndrome (IBS) in this population.

The findings of this study helped to estimate the burden of constipation among adult women living in the DRC. These findings have important clinical implications for the Democratic Republic of Congo. They could contribute to improving national health policies related to anorectal dysfunctions by raising awareness among women about constipation symptoms and available management options, encouraging them to seek medical care when needed. Moreover, strengthening the capacities of healthcare professionals in the screening and management of constipation, based on the resources available in the country, would enhance patient care. Finally, this study provides a foundation for future research to further explore aspects discussed or not addressed here, ultimately contributing to the development of evidence-based management protocols for constipation in the DRC.

### Study Strengths and Limitations

This is the first study conducted in the Democratic Republic of Congo to assess the prevalence and risk factors of constipation among adult women. It is distinguished by its large sample size and takes into account the ethnolinguistic diversity of the population. It was based on self-reported data collected through French-language questionnaires. Trained and qualified medical interpreters facilitated the process, as the questionnaires had not been validated in the national languages (Tshiluba, Kikongo, Swahili and Lingala). The absence of validated versions in the local languages spoken by the women may represent a potential source of bias. The nature of this study, focusing on the pelvic area and involving a sensitive topic, may have led some women to decline participation, which could introduce a selection bias. The absence of certain variables in this study, such as dietary habits, comorbidities, medication use and physical activity, may affect the interpretation of risk factors in this sample. Although methodology and sample size were carefully conceived, potential recall bias, cultural taboos and underrepresentation of rural women limit the full generalizability of the findings to all Congolese women.

## Conclusion

In conclusion, this study shows that the prevalence of constipation among adult women in the Democratic Republic of Congo is not negligible. This rate is similar to those observed in other international studies on constipation. The symptoms most frequently associated with constipation include hard stools, straining during evacuation, pain during straining/defecation, infrequent bowel movements and a feeling of incomplete evacuation, mainly suggestive of functional constipation and obstructed defecation, with a marked prevalence in women over 50 years.

Age, socio-cultural factors, and mental and physical health influence the prevalence of this condition. Multivariable analysis revealed that good mental health (quality of life) and belonging to the Baluba ethnolinguistic group were protective factors against constipation. Finally, it emerged that constipation also affected women’s quality of life, particularly their sexual life.

## Data Availability

The data used in this study are not publicly available for privacy and ethical reasons. However, they can be obtained from the corresponding author upon reasonable request and with approval from the ethics committee.
